# A Rare Case of Meningitis Caused by *Alcaligenes faecalis* in an Immunocompetent Patient

**DOI:** 10.1155/2022/1559360

**Published:** 2022-08-31

**Authors:** Kevin Cantillo García, Oscar Calderón Duran, Tomás Acosta Pérez, Ángel Vásquez Jiménez, Emerson Madrid Pérez, María Cristina Martínez-Ávila, Tomás Rodríguez Yánez, Amilkar Almanza-Hurtado

**Affiliations:** ^1^Intensive Care Unit, Clínica La Ermita, Cartagena, Colombia; ^2^Internal Medicine Program, Universidad Libre de Colombia, Cali, Colombia; ^3^Medicine Program, Universidad Del Sinu, Cartagena, Colombia; ^4^Epidemiology and Public Health, BIOTOXAM Group, Universidad de Cartagena, Cartagena, Colombia; ^5^Intensive Care Unit, Universidad de Cartagena, Cartagena, Colombia

## Abstract

*Alcaligenes faecalis* (*A. faecalis*) is a Gram-negative rod rarely isolated as an infective bacterium worldwide. The first cases of infections caused by this microorganism, such as pneumonia, soft tissue infections, urinary tract infections, bacteremia, and meningitis, date back more than 40 years and are almost entirely in newborns and immunosuppressed hosts. Optimal antibiotic therapy for *A. faecalis* has not been well established in the literature. We report a case of an immunocompetent patient in Colombia who had meningitis due to *A. faecalis* after a dental procedure. It is important to know about this microorganism that nowadays could be considered a potentially emerging pathogen in immunocompetent adults.

## 1. Introduction

*Alcaligenes faecalis* (*A. faecalis*) is an aerobic, nonfermentative, catalase and oxidase-positive, nonencapsulated, Gram-negative rod [1]. It is named for its ability to produce an alkaline reaction in certain media [2, 3]. *A. faecalis* is the most frequently isolated member of the Alcaligenaceae family in the laboratory [2, 3]. It could be found in the oil, water, and hospital environment, in objects such as mechanical ventilators, hemodialysis systems, and intravenous solutions [1, 4].

Although it is considered a normal saprophytic inhabitant of the human intestinal tract, it has been reported to cause dysentery and gastrointestinal infections [5–7] and has been isolated in urine, blood, wounds, feces, cerebrospinal fluid (CSF), and respiratory secretions [1].

*A. faecalis* usually causes opportunistic infections in immunocompromised hosts. In fact, it has been associated with severe infections such as peritonitis, bacteremia, endocarditis, endophthalmitis, skin and soft tissue infections, urinary tract infections, otitis media, pneumonia, and meningitis [1, 6], the latter being described mainly in neonates [8, 9].

Systemic and severe infections in immunocompetent hosts with this microorganism are very rare; there are limited data available. Herein, we present the first described case of meningitis due to *A. faecalis* in an immunocompetent adult in Colombia with a favorable outcome.

## 2. Case Description

A 30-year-old female with no relevant pathological history came to the emergency department due to seven days of fever and headache, with an intensity of 8/10 on the visual analogue scale (VAS) of pain, photophobia, and neck pain. Subsequently, this was accompanied by an acute confusional syndrome, with variations in her behavior having aggressiveness and prosopagnosia.

On physical examination, vital signs were normal: blood pressure: 107/58 mmHg, mean blood pressure: 71 mmHg, heart rate: 88 beats/minute, respiratory rate: 18 breaths/minute, oxygen saturation: 99%, temperature: 36.2°C, and glucometer: 110 mg/dl. She was in poor general condition, stuporous, with neck stiffness accompanied by a positive Kerning and Brudzinski sign, a positive jolt accentuation sign, and an 11/15 Glasgow coma scale (GCS). The presence of a periodontal abscess was striking. The rest of the physical examination was unremarkable.

At anamnesis, patient referred a persistent orodental pain which required a dental procedure fifteen days ago; she denied imaging realization and commented received an antibiotic (amoxicillin 500 mg every 8 hours) for 3 days ordered by a dentist. Blood tests reported the following: leukocytes: 4.87 × 10^3^/mm^3^, neutrophils: 52.80%, lymphocytes: 41.7%, hemoglobin: 7.7gr/dL; hematocrit: 25.4%; platelets: 361 × 10^3^/uL, glycemia 70 mg/dL, and initial chest X-ray was normal. A brain computed tomography (CT) was performed, reporting no space-occupying lesions, ischemic, hemorrhagic, and/or tomographic signs of intracranial hypertension.

With the described findings, central nervous system (CNS) infection was suspected, and due to disturbance of consciousness of fluctuating course, the patient was transferred to the intensive care unit for monitorization. Empirical therapy with broad-spectrum antibiotics (ceftriaxone 2 g every 12 hours) and corticosteroids (dexamethasone 10 mg every 6 hours) was started. Blood and urine cultures were taken.

A lumbar puncture was performed. The CSF cytochemical reported the following: glucose in liquid 58 mg/dl, proteins in liquid 70.80 mg/dl, colorless, appearance: transparent, pH: 8, density: 1000, leukocytes: 8.24 × 10^3^/mm^3^, polymorphonuclear cells: 60%, and mononuclear cells: 40%. A nonreactive nontreponemal test (VDRL), negative-staining India ink, and negative CSF culture for *M. tuberculosis* were obtained. Also, blood and urine cultures were reported as negative without any bacterial growth.

CSF culture reported single isolation of multisensitive *A. faecalis* (Table 1).

Considering the rareness of the microorganism isolated in an adult without obvious risks for this infection leads to concern that the single CSF culture isolate may have been a contaminant. Thus, the culture was repeated, yielding the same result, confirming the diagnosis of meningitis due to *A. faecalis*. The patient was assessed by infectious diseases, who recommended giving antibiotic management for 14 days.

Given favorable evolution, improvement in the state of consciousness, and GCS, she was transferred to the hospitalization ward to continue treatment for the next seven days. After completing antimicrobial coverage, the patient was discharged without sequelae or neurological repercussions.

## 3. Discussion

*A. faecalis* is an etiological agent infrequently cited in the literature as the cause of systemic infection in adults. It has been isolated in different clinical scenarios, and medical equipment and solutions have been found to be contaminated with this bacterium [1, 5]. This microorganism is recognized as an opportunistic pathogen responsible for serious infections in immunosuppressed hosts, newborns, and infants. Nevertheless, CNS infections are rare. In our case, it was not possible to demonstrate significant comorbidities or immunosuppression [5, 8, 9], which makes this case relevant.

The published information is limited. The first reported literature dates back to case reports over 50 years old, primarily for pediatric patients [5, 8, 9]. Cases of hematogenous dissemination and meningitis derived from a case of dysentery in a 3-year-old infant, atypical presentations of septicemia and meningitis in newborns, and even hydrocephaly in a newborn are highlighted [5, 8, 9]. We could not identify extensive cases of meningitis due to this causal agent in immunocompetent adults.

Additionally, the most extensive case series of infections due to *A. faecalis* reported in the literature included 191 cases, of which 45 were bacteremia, 36 were cystitis, 23 were skin and soft tissue infections, 23 were otitis media, and 16 were meningitis [1]. It is worth to mention that we do not find evidence of Colombian cases in this retrospective analysis [1], considering this case the first report in this regard.

Most isolations of *A. faecalis*, identified in blood or respiratory secretions, are related to contamination of hospital fluids or equipment, promoting colonization of the human host [4]. Despite that, when there are symptoms, it is important to rule out the route of infection and the associated organ compromised [6].

The mechanisms of inoculation of this bacterium are multiple. However, the symptomatic disease involves a complex interplay between the virulence factors of the pathogens and the host's immune response. Weinstein and Wasserman describe a series of cases of *A. faecalis* infection, among which they report cases of meningitis after CNS procedures and trauma [6]. Within this description, infections related to skull fracture, meningocele correction, or acute otitis media stand out [6]. In this case, it is believed that the pathogenesis and pathophysiology of meningitis were the results of previous colonization of mucous membranes that, with the patient's dental procedure, favored the invasion of the bloodstream from the maxillary alveolar bone with subsequent hematogenous dissemination where the microorganism crossed the blood-brain barrier into the CSF.

It should be added that meningitis by *A. faecalis* and its clinical manifestations are not different from other CNS infections, a reason to why the patient presented with positive Kerning and Brudzinski sign, a positive jolt accentuation sign. In this case, we rule out other more frequent microorganisms that caused meningitis before the final diagnosis and discard that the microbiological isolation was due to contamination because the patient had symptoms and two CSF cultures with single isolation of *A. faecalis.*

The optimal antibiotic therapy for *A*. *faecalis* has not been well established in the literature and is often difficult to treat due to its increased resistance to several antibiotics [1, 3]. The *A. faecalis* isolated in our case was multisensitive, which allowed the use of conventional antibiotics that cross the CNS to obtain a favorable response with no neurological sequelae.

## 4. Conclusions

Our manuscript's importance lies in the lack of scientific literature regarding cases of meningitis due to *A. faecalis* in immunocompetent adults and in the fact that it is the first case described in Colombia. In conclusion, meningitis due to *A. faecalis* is rare and usually occurs in newborns or infants. However, it has also been described in adults, our case being one of the few in the literature. It is important to know about this microorganism that nowadays could be considered a potentially emerging pathogen in immunocompetent adults.

## Figures and Tables

**Table 1:** Results of antibiogram culture of CSF.

Antimicrobial agent	*Alcaligenes faecalis*
Amoxicillin	S ≤ 16
Aztreonam	R > 16
Ceftazidime	S:8
Ceftriaxone	S:8
Cefepime	S:8
Gentamycin	S ≤ 4
Imipenem	S ≤ 1
Meropenem	S: ≤ 1
Piperacillin/tazobactam	S: ≤ 16
Tobramycin	S:<04

S, sensitive; R, resistant.
